# Enabling Energy Harvesting-Based Wi-Fi System for an e-Health Application: A MAC Layer Perspective

**DOI:** 10.3390/s22103831

**Published:** 2022-05-18

**Authors:** Golshan Famitafreshi, Muhammad Shahwaiz Afaqui, Joan Melià-Seguí

**Affiliations:** 1 Internet Interdisciplinary Institute (IN3), Universitat Oberta de Catalunya (UOC), Rambla del Poblenou, 154, 08018 Barcelona, Spain; gfamitafreshi@uoc.edu; 2 School of Computing, Engineering & Digital Technologies, Teesside University, Middlesbrough TS1 3BX, UK; m.afaqui@tees.ac.uk; 3 Faculty of Computer Science, Multimedia and Telecommunication, Universitat Oberta de Catalunya (UOC), Rambla del Poblenou, 156, 08018 Barcelona, Spain

**Keywords:** energy harvesting, e-Healthcare, Wi-Fi technology, MAC layer, optimization, MIoT, contention window, sleep mode, objective function

## Abstract

The adverse impacts of using conventional batteries in the Internet of Things (IoT) devices, such as cost-effective maintenance, numerous battery replacements, and environmental hazards, have led to an interest in integrating energy harvesting technology into IoT devices to extend their lifetime and sustainably effectively. However, this requires improvements in different IoT protocol stack layers, especially in the MAC layer, due to its high level of energy consumption. These improvements are essential in critical applications such as IoT medical devices. In this paper, we simulated a dense solar-based energy harvesting Wi-Fi network in an e-Health environment, introducing a new algorithm for energy consumption mitigation while maintaining the required Quality of Service (QoS) for e-Health. In compliance with the upcoming Wi-Fi amendment 802.11be, the Access Point (AP) coordination-based optimization technique is proposed, where an AP can request dynamic resource rescheduling along with its nearby APs, to reduce the network energy consumption through adjustments within the standard MAC protocol. This paper shows that the proposed algorithm, alongside using solar energy harvesting technology, increases the energy efficiency by more than 40% while maintaining the e-Health QoS requirements. We believe this research will open new opportunities in IoT energy harvesting integration, especially in QoS-restricted environments.

## 1. Introduction

The Internet of Things (IoT) ecosystem includes a massive number of physical devices, which interact through the Internet to improve and enhance various applications and services [1]. According to the Cisco Annual Internet Report [2], by the end of 2023, the number of connected IoT devices will increase to 29.3 billion devices. Thus, conventional batteries, known as the most common energy source for IoT devices, might not be efficient. This inefficiency is due to the limited lifetime of conventional batteries, which require frequent replacement and maintenance. The adversity of battery replacement and maintenance intensifies, especially where the devices are placed in hard-to-reach areas and dangerous places. In addition, the disposal of this amount of batteries releases toxic material into the environment.

Different passive techniques have been introduced in the literature to diminish the disadvantages of conventional batteries and reduce their maintenance cost. One of these techniques is deploying energy harvesting technologies, which is considered a promising solution to provide enough energy for IoT devices and keep them powered up.

Although all IoT sectors benefit from energy harvesting technologies, deploying these technologies in Medical IoT (MIoT or IoMT) offers a double benefit: reducing the maintenance cost and saving human life. This would be especially beneficial in the event of a pandemic, such as the one that occurred in 2020 (the COVID-19 crisis), when hospitals’ total capacity was nearly fully occupied by patients who required specialized care [3]. In these cases, establishing a center such as a field hospital or a mobile medical unit is inevitable. Since these constructions face new challenges in providing enough reliable energy sources for the monitoring devices, cooperating energy harvesting technologies (solar cells or Piezoelectric harvesters) can help deliver sustainable and reliable energy sources [4] for these temporary medical centers.

Another critical challenge in mobile unit deployments is selecting relevant wireless communication technologies. Among all the available wireless communication technologies, Wi-Fi is considered to be cost-effective and accessible deployment technology. Moreover, the Institute of Electrical and Electronics Engineers (IEEE)-based Wireless Local Area Network (WLAN) standard [5] has been widely used in different environments and is one of the most successful wireless communication technologies for indoor environments. For instance, in the case of the coexistence of Wi-Fi and Zigbee in indoor environments, since Wi-Fi devices have a shorter Channel Clear Assessment (CCA) time, they have priority over Zigbee devices. In addition, since Zigbee frame transmissions have a longer frame in the air time, they suffer more than Wi-Fi devices from the hidden node problem [6]. Furthermore, IEEE 802.11 introduces amendments such as IEEE 802.11ax [7], IEEE 802.11be (not standardized yet) suitable for dense indoor environments, and IoT networks similar to a mobile medical unit.

Wi-Fi technology offers powerful benefits for deploying in dense indoor environments. However, the issues inherent to traditional Wi-Fi networks could be intensified in these environments. The challenges that dense Wi-Fi networks face can be studied from two perspectives, the physical layer and the MAC layer. One of the immediate issues related to the physical layer is the placement of the APs in the dense network, which causesoverlapping coverage and have a significant effect on the spectrum efficiency and throughput of the network [8]. Regarding the MAC layer perspective, the other issue is channel interference due to the high number of devices, originating due to the contention-based nature of the MAC layer of IEEE 802.11. In these environments, channel interference causes exposed and hidden node problems and increases the collision rate [9,10]. One of the consequences of all these issues is the increase in the systems’ energy consumption.

As we highlighted, due to the high energy-consuming nature of MAC layer operations and the challenges that it faces, integrating an energy harvester may not be sufficient to keep the MIoT devices powered up. Therefore, to reach a sustainable MIoT system without degrading the system’s performance and maintaining the QoS at a certain level, there is a need to optimize the energy consumption of the Wi-Fi communication technology at the MAC layer and adapt it to the MIoT systems.

To address the integration of energy harvesting technologies within a dense Wi-Fi network, in this paper, we propose an AP coordination-based optimization algorithm (inspired from the AP coordination method under discussion in the upcoming IEEE 802.11be amendment), that supports the QoS requirements for a restricted QoS environment while mitigating the network’s energy consumption. Additionally, we implement a sleep/wake-up method, which considerably reduces network energy consumption. The proposed algorithm is evaluated under extensive simulations in a dense Wi-Fi network in a field hospital, where all the devices are equipped with solar cells. To the best of our knowledge, this is the first time the suggested combination of AP coordination-based and sleep/wake-up algorithms has been outlined in the literature to minimize network energy consumption while preserving a specific degree of QoS for a solar-based dense e-Health environment. Furthermore, we propose an innovative objective function used for evaluation proposes. To summarize, this paper includes the following contributions:

- We conduct extensive simulations in the Network Simulator 3 (ns-3) environment, which can accurately mimic the deployment of Wi-Fi communication for solar-based medical devices in the proposed scenario.
- We incorporate the AP coordination idea from the upcoming IEEE 802.11be standard in our AP coordination-based optimization approach, while also maintaining backward compatibility with the IEEE 802.11 standard.
- We propose an objective function based on medical-grade QoS criteria and energy usage.
- We propose a sleep/wake-up mechanism that puts non-AP stations to sleep for a time interval if residual energy falls below a particular threshold. This approach allows network energy consumption reduction while maintaining the desired level of QoS.

The remainder of this paper is organized as follows. In Section 2, the main concepts around the fundamental IEEE 802.11 MAC layer mechanisms and the relevant amendments, energy harvesting technologies, and e-Health applications are introduced. Section 3 highlights the relevant existing works in the literature. In Section 4 and Section 5, the applied methodology and all the steps taken for the simulations are explained. Section 6 provides the performance evaluation of the AP coordination-based optimization algorithm, along with an analytical discussion. Finally, in Section 7, some final remarks and future directions are given.

## 2. Background Study

In this section, we divide the main concepts of this paper into three parts, briefly explain each concept and then find their intersection points. These concepts are listed as electronic healthcare (e-Healthcare), IEEE 802.11 (Wireless communication technology), and the relevant energy harvesting technologies for the e-Healthcare use case.

### 2.1. E-Healthcare

E-Healthcare refers to the deployment of information and communication technologies (ICTs), such as IoT, cloud computing, big data, and artificial intelligence, to intelligently manage the healthcare system and make it on-demand, more accurate, and more efficient [11,12,13]. E-Health enables versatile telehealth services (telemedicine, telesurgery, telerehabilitation), wearable devices, e-Health records, smart healthcare applications, etc. These services improve patient monitoring for medical staff and patients, facilitate self-health management, and encourage people to form healthier habits. In addition, e-Health reduces human error and the cost of activities simultaneously.

As explained in the introduction, the growing trend of the MIoT as a subcategory of IoT faces various challenges at different levels of its architecture. As the first level of the MIoT architecture, the sensing and perception layer includes real hardware and is responsible for collecting patients’ data. At this level, the devices need to be low-power and low-cost, small in the physical dimension, and user friendly. Since medical sensors have to provide a long operational lifetime, having batteries with a limited lifespan is challenging and motivates the use of low-power consumption devices or even devices without batteries. Removing the battery from MIoT increases the flexibility of wireless devices and the operational lifetime of medical devices, which is especially vital for hard-to-reach devices and reduces maintenance costs.

The second level defines the communication protocols regardless of the use of either wired or wireless communications. At this level, different power management and optimization mechanisms can be applied to satisfy the devices’ required low-power consumption feature. In addition, since the content of data transmitted in MIoT is privacy-sensitive, network security features such as confidentiality, integrity, and availability of medical data are challenging. Moreover, since MIoT is considered a QoS-restricted environment, especially in terms of the Packet Loss Ratio (PLR) and delay [14], at this level, ensuring the medical-related QoS requirement is a demanding issue.

The last level is responsible for managing and controlling the applications on devices, which the medical business providers control. Furthermore, some information technologies such as artificial intelligence, deep machine learning for healthcare, and big data belong to this architectural level of MIoT. At this level, the user’s privacy is a challenging issue [4,15].

**Figure 1:**
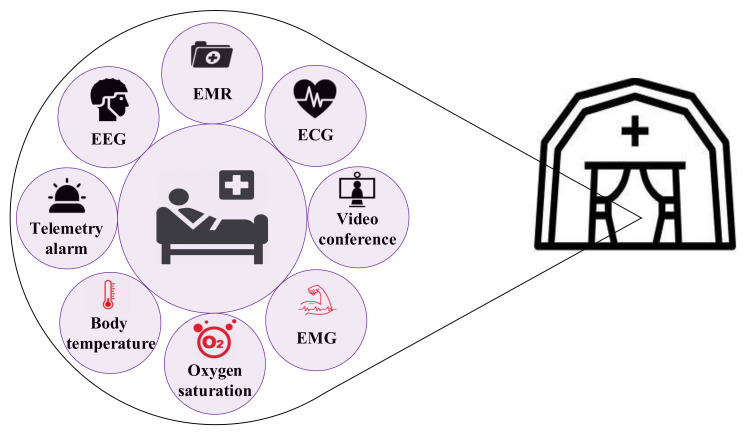
An example of MIoT applications in a field hospital.

Figure 1 demonstrates a patient who is located in a field hospital and is connected to different medical sensors (the applications which are in red are not considered in the simulation setup), and other real-time applications such as video conferencing with a doctor.

### 2.2. IEEE 802.11

The IEEE 802.11 working group has been standardizing different amendments by specifying various sets of MAC and physical layers for WLAN communications.

**Figure 2:**
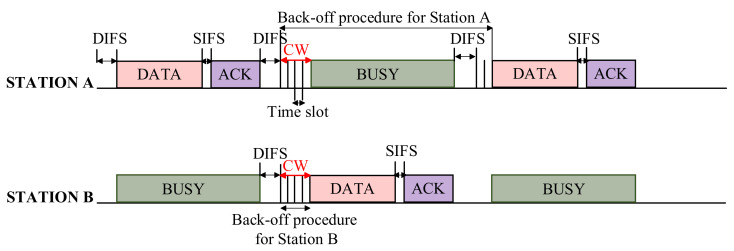
CSMA/CA Back-off procedure.

The fundamental mechanism of the MAC layer in IEEE 802.11 standard is known as the Distributed Coordination Function (DCF). It uses a Carrier Sense Multiple Access/Collision Avoidance (CSMA/CA) method with binary exponential back-off. Figure 2 demonstrates the default access technique known as a two-way handshaking scheme. According to CSMA/CA mechanism, stations monitor the channel before sending the data frame. They will start a back-off countdown if they sense the channel idle for a specific time interval known as Distributed Inter-Frame Space (DIFS). Otherwise, if the channel is sensed as busy, the stations keep monitoring the channel until the channel is sensed idle for a DIFS. Then, the back-off countdown timer starts after the channel is sensed idle for a DIFS. Since DCF is defined in a discrete-time back-off manner, each transmission must begin at the start of the time slot.

The back-off procedure is started by initializing the Contention Window (CW) to CW${}_{\min}$, where the station chooses a random number within (0, CW-1). The counter decreases the back-off timer if the channel is sensed idle during a time slot. However, in the case of data frame transmission, the timer halts and only reactivates if the channel stays idle for more than DIFS. If the data frame is unsuccessful, the CW is doubled until it reaches its maximum value ($2^{n}$CW${}_{\min}$ = CW${}_{\max}$). Once a data frame is transmitted, the sender waits for an Acknowledgment (ACK) frame to confirm the data frame’s correct reception. Suppose the station that started the transmission does not receive an ACK frame during the ACK timeout period. In that case, it understands that a collision happened. Therefore, the station retransmits the data frame according to the back-off process. The data frame will be discarded if it experiences more collisions than the maximum retry limit.

The IEEE 802.11 standard group defines another mechanism known as Enhanced Distributed Channel Access (EDCA), which supports differentiated Quality of Service (QoS) in Wi-Fi communications. This mechanism introduces four different Access Categories (AC${}_{\mathrm{VO}}$, AC${}_{\mathrm{VI}}$, AC${}_{\mathrm{BE}}$, and AC${}_{\mathrm{BK}}$) to prioritize channel access, where the AC${}_{\mathrm{VO}}$ has the highest priority and AC${}_{\mathrm{BK}}$ has the lowest priority. The AC${}_{\mathrm{VO}}$, AC${}_{\mathrm{VI}}$, AC${}_{\mathrm{BE}}$, and AC${}_{\mathrm{BK}}$ categories are meant for voice, video, best-effort, and background traffic. According to this mechanism, the MAC layer parameters such as CW${}_{\min}$ and CW${}_{\max}$, Arbitrary Inter-Frame Space (AIFS), Transmission Opportunity (TXOP), and queue length are set to different values to achieve this prioritization. For instance, AC${}_{\mathrm{VO}}$ parameters are assigned to the smallest values among other categories to give the highest transmission opportunity to the traffic under this category. However, since different applications require various ACs, and Wi-Fi proposed fixed EDCA parameters for each AC (Table 1), it is unsuitable and unfeasible for heterogeneous networks [16], such as e-Health networks, where the time-sensitive and emergency traffic require a certain level of QoS. For this reason, new ACs with special queues are required. Moreover, as we explained, since the CW is the principal parameter of the back-off procedure, among the EDCA-related parameters [17], which are listed in Table 1, CW has the most impact on rescheduling the transmissions and QoS parameters.

As highlighted in previous works [18,19,20], since the inherent behavior of DCF and EDCA mechanisms are contention-based, collisions may be caused by simultaneous transmissions, which is one of the reasons that imposes extra energy consumption on the Wi-Fi stations. It is worth mentioning that, in Time Division Multiple Access (TDMA), a control channel makes the channel collision-free; however, this feature is not available on Wi-Fi. The other reason behind the energy-hungry feature of the DCF mechanism is the transmission errors due to the imperfect channel condition, which causes re-transmission. Besides the amount of energy consumed in the transmission state, the idle state of DCF can also consume a significant amount of energy. Although various methods have been introduced to reduce these effects, they need to precisely select the involved parameters to avoid extra energy consumption [21]. For instance, setting the beacon and idle intervals in the power-saving mode is very important to prevent frequent wake-up nodes or simultaneous wake-ups from wasting the station’s energy.

#### 2.2.1. Previous IEEE 802.11 Amendments

The IEEE 802.11 standard group introduced different features to the amendments to meet the IoT requirements while reducing the energy consumption of the MAC layer operations. For instance, IEEE 802.11ah [22] provides some additional features to the MAC layer of IEEE 802.11, such as hierarchical Association IDentifiers (AID), group sectorization, Restricted Access Window (RAW), Relay AP, bi-directional TXOP, and Target Wake Time (TWT) [23], that make this amendment enable supporting the IoT concept. However, IEEE 802.11ax and IEEE 802.11ba [24] were designed to support dense and low-power consumption deployments, respectively. The Basic Service Set (BSS) coloring MAC feature in IEEE 802.11ax makes this amendment suitable for dense network deployment [9]. Furthermore, the energy efficiency is provided through microsleep, TWT, and Opportunistic Power Save (OPS) mechanisms [25]. In contrast to these two amendments, IEEE 802.11ba balances the trade-off between low-power consumption and latency by implementing the concept of Wake Up Radio (WUR) [26].

#### 2.2.2. Wi-Fi 7

**Figure 3:**
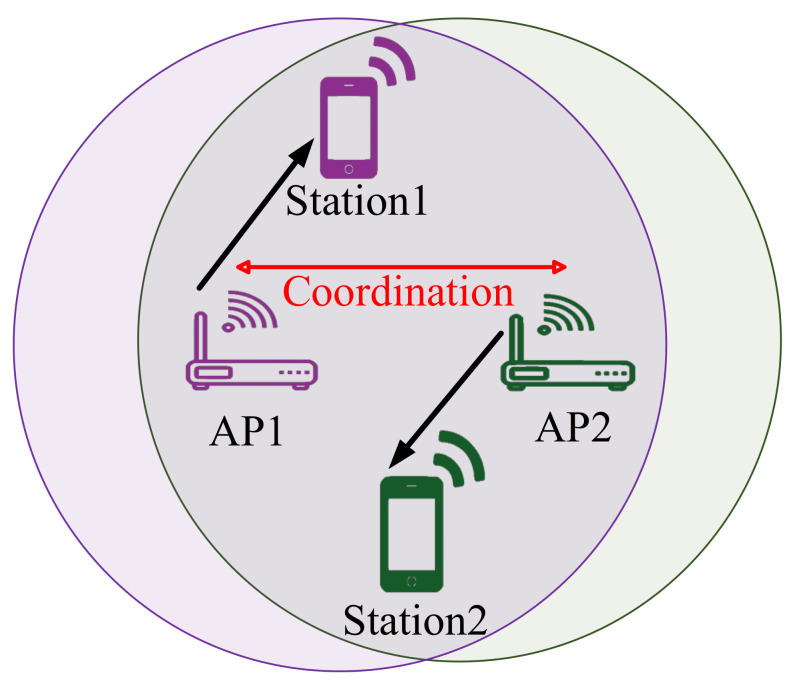
Wi-Fi 7 allows AP coordination.

Along with the aforementioned IEEE 802.11 amendments, an upcoming IEEE 802.11be has features that IoT systems can benefit from them. The IEEE 802.11be is built on top of the IEEE 802.11ax amendment and will support real-time applications, where QoS provisioning is challenging. In addition, this amendment will provide a very high data rate and makes massive Multi-Input Multi-Output (MIMO) communications possible. Some advanced modifications and enhancements are introduced at the Physical (PHY) and MAC layers to fulfill these features. For instance, the AP coordination and Hybrid Automatic Repeat Request (HARQ) are presented at the MAC layer. According to the AP coordination technique, so-called master APs, to improve the performance of their associated non-AP stations, have the ability to communicate with other APs located within its transmission range (slave APs), where the master AP receives the beacon frames the slave APs. In this technique, the master AP is able to dynamically request the slave APs to reschedule the resources based on the channel conditions (cf. Figure 3) [27]. It is worth mentioning that this technique is specifically designed for the needs of uncoordinated systems; however, the coordinated systems can benefit from the concept of this technique. Moreover, the HARQ technique combines the forward error correction method and ARQ to deliver reliability for data frame transmission. Furthermore, the pick rate, channelization, and time planning at the PHY layer are improved [28,29].

### 2.3. Energy Harvesting in E-Healthcare

MIoT can benefit from different types of harvesting technologies to keep power-up MIoT devices and introduce battery-less devices [30,31,32,33]. Among the existing energy harvesting mechanisms, photo-voltaic, piezoelectric, Thermoelectric Generator (TEG), and Radio Frequency (RF) are the most relevant technologies for the MIoT [34,35].

Photo-voltaic cells absorb the energy from artificial light or sunlight and then converts it to electric energy. Based on the amount of light radiation, the power density of the cell varies from 10 µW/${\mathrm{cm}}^{3}$ to 100 mW/${\mathrm{cm}}^{3}$ [4].

A rectenna or RF harvester captures the RF signals (dedicated or radiated signals), and then the rectifier circuit (peak detector and voltage elevator) converts them to DC signals. Depending on the physical features and position of the Wireless Energy Harvester (WEH), the power density of RF harvesters varies from 0.1 µW/${\mathrm{cm}}^{2}$ to 300 µW/${\mathrm{cm}}^{2}$ [4].

TEG or thermocouple captures the generated voltage based on the temperature difference between the two types of metals or semiconductors. Various types of the TEG provide a wide range of power density from 40 µW/${\mathrm{cm}}^{2}$ to 50 mW/${\mathrm{cm}}^{2}$.

A piezoelectric energy harvester obtains energy from a crystal-ionized piezoelectric material under a certain strain (human motion and activity). This harvester converts kinetic energy to electric energy. Depending on the harvester material and the amount of kinetic energy, the piezoelectric power density varies from 0.021 µW/${\mathrm{mm}}^{3}$ to 2 W/${\mathrm{cm}}^{3}$ [4].

Due to the high power density of solar cells and piezoelectric harvesters and their form factor flexibility, these harvesters are widely used in IoT applications [4]. However, in MIoT systems, since TEG and piezoelectric harvesters are able to harvest energy from the human body, they attract much attention from industry and academia. The aforementioned energy harvesters are summarized in Table 2.

**Figure 4:**
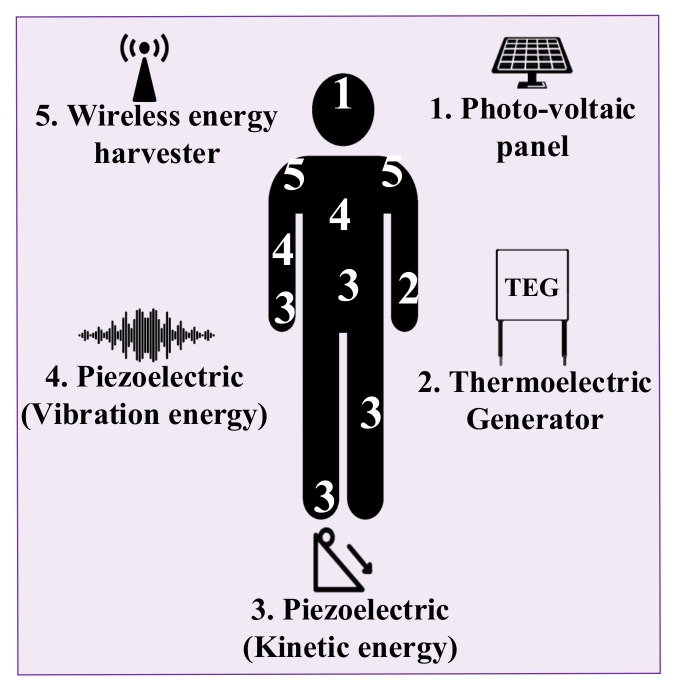
An example of the energy harvesters placement on the human body in the MIoT system.

Figure 4 represents the relevant energy harvesting technologies that can be implemented in an e-Healthcare sector based on the positions and activities of the patient. The possible positions on the human body for each energy harvester are determined by a number corresponding to that specific energy harvester technology. For instance, a kinetic-based energy harvester (piezoelectric) is able to harvest energy from the movement of the ankle, finger, or foot of a person, while a vibration-based energy harvester can be placed on the chest or elbow of the person. These points are depicted as numbers 3 and 4 [36] in Figure 4, respectively. Since the photo-voltaic panel needs to be in contact with artificial light or sunlight radiation, the most suitable position for this energy harvester is the forehead of the person (number 1) [37]. Although the WEH does not need a direct connection to the wireless waves, one of the suitable positions for its placement is on the shoulder of the person (number 5) [38]. Finally, the wrist of the person can be a proper position for TEG harvester placement. Since in the wrist measuring the temperature difference between the human body and the air can be more feasible (number 2) [39].

## 3. Related Work

In recent years, IEEE 802.11 WLAN has been one of the attractive wireless technologies deployed in IoT networks. However, due to the high interference nature of its medium and high energy-consuming MAC layer operations, deploying an energy harvesting technology to extend the lifetime of the IoT devices while ensuring QoS features becomes a challenging issue. This issue becomes more demanding for QoS-restricted environments, where real-time, multimedia and distributed emergency applications are deployed, such as the healthcare sector, disaster recovery, and industrial emergency traffic. Although there have been many works in the literature on QoS requirements provisioning, they do not consider the requirements of energy harvesting technology deployment aligned with the QoS guarantee as a critical point in the IEEE 802.11 WLAN. Thus, to completely understand this issue, there is a need to have a structural literature review of MAC layer modifications to provisioning QoS and the integration of energy harvesting technologies with the IEEE 802.11 standard. This section will explain the related works regarding these perspectives.

### 3.1. MAC Layer Modification

In this subsection, we explain the relevant studies on MAC modification and enhancement to meet the QoS requirements within the IEEE 802.11 standard.

In 2005, the IEEE 802.11e working group introduced the IEEE 802.11e amendment, whose MAC layer supports time-sensitive applications [17]. However, the EDCA mechanism in this amendment has limitations and does not support QoS-restricted environments. One of the studies on enhancing IEEE 802.11e QoS is proposed in [40]. In this work, the authors consider three types of traffic, real-time medical traffic such as Electrocardiogram (ECG), emergency alarms, and non-real-time traffic. The authors assign different levels of priority to these applications. Then, based on the QoS requirements and the level of priority of each medical application, they introduce an adaptive AIFS algorithm for each medical traffic. In this algorithm, the station with the higher priority traffic maintains the required QoS level by requesting the stations with lower priority traffic to increase their AIFS values and delay their transmissions. In addition, they propose an admission control algorithm, which is able to guarantee the QoS for the highest priority traffic. Although the authors show that the proposed algorithms perform well under saturated conditions where more stations join the network, the QoS may not be guaranteed.

As we explained in Section 1, compared to the AIFSN parameter, the CW value is another MAC layer parameter that has a more significant impact on the network performance metrics such as the end-to-end delay, throughput, PLR, collision rate, and even energy consumption of the network. For this reason, extensive research has been conducted on CW value variations to address QoS restrictions. In some works, the CW size is fixed at an optimal value. In contrast, in some other research, the CW size is dynamically adapted to an optimal value regarding the network conditions. Tian et al. in [41] proposed an algorithm based on the CW value doubling not only when a collision occurs but also when the channel is sensed as busy. By doubling the CW value, the stations which suffer from overhearing can benefit more by reducing their collision rate. However, the stations with less overhearing will face longer end-to-end delay. In contrast to the previous work, to mitigate the long delay, Syed et al. in [42] proposed a dynamic CW adaptation based on the network load. The proposed algorithm estimates the number of active stations to reduce the number of retransmissions due to the high collision rate in high traffic load conditions. Then it selects the most proper CW value based on that estimation for each access category. Thus, for a dense network, a higher value of CW is determined, and a lower CW value is specified for lower traffic loads. Therefore, this algorithm improves the throughput and collision rate for the delay-sensitive application while minimizing the delay of the network. However, in these studies, the energy efficiency of the network was not considered.

Accompanied by the AIFS and CW dynamic adaptations, in the IEEE 802.11ah amendment, the QoS can be guaranteed through the RAW feature. However, this approach has not received much attention. The authors in [43] assign different channel access timing for each group of stations based on the priority of their QoS requirements. For this reason, they define two types of stations, stations with periodic traffic and stations with non-periodic traffic. In the case of frame collision, the periodic stations halt the transmission procedure, and only non-periodic ones continue to transmit. Based on this algorithm, the stations with higher priority have more chance to access the channel than stations with lower priority. This means that the QoS in the stations with the lower priority may not be guaranteed. In another QoS provisioning study, the authors mathematically model the EDCA concept into the RAW feature of IEEE 802.11ah [44]. This algorithm performs station categorization based on the back-off value, the idle state probability, and the throughput during the non-idle states. However, according to the proposed algorithm, although QoS is guaranteed in scenarios with a low traffic load, the stations with lower priority will suffer from long delay and low throughput. To address this issue, the authors in [45] introduce a longer RAW size for stations with higher priority compared to the lower priority stations. Nevertheless, the proposed algorithm is not completely backward-compatible with IEEE802.11ah due to its back-off procedure.

One of the latest IEEE 802.11 amendments, known as IEEE 802.11ax, improves the concept of multi-user transmission by introducing Orthogonal Frequency-Division Multiple Access (OFDMA). This feature makes channel scheduling and resource allocation flexible for high-density networks. In [46], an efficient channel scheduler is implemented in the AP, which is able to increase the resource unit allocation. The algorithm makes the decision based on the amount of data and the information priority of the QoS in each associated station. Therefore, the proposed algorithm provides the QoS requirements in dense networks.

In recent years, with the advent of the concept of artificial intelligence, researchers have tried to benefit from different machine learning methods in the e-health sector to improve the performance of the healthcare systems, such as telehealth monitoring and remote patient monitoring. Malche et al. [47] propose a machine learning method for a MIoT device that performs real-time monitoring of the vital signal of the patient during specific activities such as walking, running, exercising, and sleeping. Although the wireless communication technology in this work is considered Bluetooth Low Energy (BLE), their work could be adapted by including the concept of master-slave communication. In addition, the sleep/wake-up method and the integration of an energy harvester can be introduced to the network. The authors in [48] propose a machine learning approach to predict the patient’s health status in real time by monitoring vital signals. However, the role of energy harvesters and methods to reduce the network’s energy consumption have not been taken into consideration.

### 3.2. Integration of the Energy Harvesting Technologies with Wi-Fi

Comprehending the current research on MAC layer modifications for QoS-restricted environments demonstrates that the MAC layer operations may consume more energy under these conditions. In addition, applying machine learning methods increases the computational complexity of the systems, and the devices’ energy consumption rises in consequence. In these cases, integrating energy harvesting techniques becomes essential. Thus, there is a need to study the integration of these technologies within the IEEE 802.11 standard. This study will lead us to elaborate on the existing gap in the literature.

One of the earliest investigations on the integration of energy harvesting technologies within the IEEE 802.11 standard is proposed in [49]. In this work, a CO${}_{2}$ sensor that communicates based on the IEEE 802.11 standard is powered up with indoor light radiation. Although the authors demonstrate the possibility of sustainable wireless communication, they do not consider energy efficiency in their experiments. They claim that the consumed energy can be reduced to half of the current value by applying an energy-efficient MAC layer protocol. The authors in [50] proposed an algorithm based on the 802.11 power-saving mechanism, which offers more priority to the stations with a lower level of energy. Each ambient energy-based station is frequently sent to the sleep mode to save energy in this algorithm. Although the proposed algorithm reduces the overhearing issue, it suffers from a long delay due to random sleep duration and wake-up modes.

Among all energy harvesting technologies, since RF harvesters can directly harvest Wi-Fi signals from nearby Wi-Fi devices, the integration of the RF harvesters with IEEE 802.11 has attracted more attention in academia and industry. For instance, in [51], the authors design, optimize and fabricate a rectenna, which is able to harvest energy from the 2.4 Ghz frequency band from Wi-Fi devices. Based on the simulation and experimental results, they demonstrate that the proposed rectenna is low-cost and easy to integrate with IoT devices. One of the latest investigations on RF harvester use in WLAN scenarios is presented in [52], in which the wake-up receiver and duty cycle concepts are combined to address the energy efficiency issue in IEEE 802.11-based communications. This work demonstrates the feasibility and flexibility of the wake-up signal to reduce the energy consumption of the uplink and downlink wireless communications. The authors claim that the proposed approach outperforms the IEEE 802.11 power-saving mechanism and can be further deployed in batteryless IoT devices.

### 3.3. Energy Harvesting MAC Layer Protocols

In addition to the works mentioned previously, there are studies in the literature on energy harvesting MAC protocols, in which the authors proposed MAC mechanisms to reduce energy consumption by balancing the trade-off between collision rate and overhead reduction, QoS provisioning, or idle listening duration. For instance, the authors in [53] provide channel prioritization based on the content of a frame while adjusting the wake-up duration to the energy level of an individual node to reduce the energy consumption of the network. The shortcoming of this method is its random back-off procedure, where nodes waste a considerable amount of energy during the long idle listening. Another QoS MAC protocol which is defined in [54], provides four different data prioritization. In this mechanism, transmissions are organized by the receiver based on each node’s waiting time, duration, and energy level. As with the previous research, the prioritization in this protocol is determined by the frame contents of each node. This protocol reduces the delay of the networks with dynamic traffic load. However, this protocol may face a long delay in applications with a high collision rate and waste the energy of the network. The first work deploys a generic energy harvester, whereas the second study integrates a solar panel to the sensor nodes. The MAC mechanisms, which are specifically designed for Radio Frequency energy harvester, are proposed in [55,56,57], where the prioritization of the frame transmissions are scheduled based on the residual energy, energy harvesting rate, or Energy Request frame (ER) of the stations. However, since these works revamp the structure of the CSMA/CA mechanism, the compatibility and adaptability of these MAC mechanisms with the IEEE 802.11 becomes a problem and requires an accurate justification [55]. Moreover, the key role of the QoS metrics provisioning is clearly defined in works such as [53,54]. In addition, integrating the energy harvesters with MAC mechanisms in these works may not lead to the reduction of energy consumption of the network.

According to the available state-of-the-art, although some simulators such as QualNet, Cooja, ns-2, and MATLAB have been widely used in IoT network simulations for analytical analysis, the energy models reveal different limitations. For instance, these models for energy harvesting systems are abstract and straightforward and do not address many energy harvesting technologies and process features. However, since the defined energy model in ns-3 is an accurate model [58], it is able to provide a simulation environment that addresses these constraints accurately. In addition, it has the ability to enable real applications through Direct Code Execution and packet sending over actual Network Interface Cards (NICs) to testbeds.

Furthermore, the impact of using energy harvesting technologies on the network’s energy consumption and QoS-restricted environments has not been thoroughly studied. Thus, in this paper, we fill this gap in the literature by applying an AP coordination optimization in a solar-based dense Wi-Fi network in the ns-3 environment. Then we introduce a sleep/wake-up duration to reduce the network’s energy consumption while maintaining the QoS metrics such as delay and PLR. Finally, we demonstrate the feasibility and energy efficiency of integrating solar energy harvesting technology in a dense medical Wi-Fi network for medical-grade QoS IoT applications based on extensive simulations and the proposed objective function. Table 3 emphasizes the originality of the proposed work by comparing it with relevant previous works.

## 4. Methodology

In this section, first we define the implementation of the proposed optimization algorithm and sleep/wake-up mode in ns-3. Then we describe the structural layout of a station in this simulator, and finally, we explain the network evaluation parameters based on their respective expressions.

### 4.1. AP Coordination-Based Optimization Algorithm

In this subsection, first we express the method that we define to find the combination of CW${}_{\min}$ and CW${}_{\max}$ for each AC, then we explain the functionality of the proposed algorithm in detail.

(1) $${\mathrm{CW}}_{\max-\mathrm{new}}={\mathrm{CW}}_{\min}+\alpha$$

Equation (1) demonstrates the method to obtain the new CW${}_{\max}$ values. In our approach, the relation between CW${}_{\max}$ and CW${}_{\min}$ maintains the same as the DCF mechanism which is given by CW${}_{\max}$= $2^{n}$CW${}_{\min}$. However, we add an initial value as α to this relation to increase the buffer slightly. In this Equation, α is the difference between the standard CW${}_{\min}$ and CW${}_{\max}$ values and defines the CW size. The α value is defined as 8, 16, and 922 for AC${}_{\mathrm{VO}}$, AC${}_{\mathrm{VI}}$ and AC${}_{\mathrm{BE}}$, respectively. According to this method, the CW changes are adapted to the traffic while maintaining the CW size constant defined in the IEEE 802.11 standard Table 1.

As explained in Section 2, in the AP coordination technique, the master APs have the possibility to communicate with slave APs to reschedule their resource allocation. This technique addresses the QoS requirements for QoS-restricted applications, particularly for real-time, multimedia, and emergency applications. Our proposed AP coordination-based optimized algorithm, divided into two main parts, is retrieved from this technique to meet the medical-grade QoS requirements while adapting energy harvesting technology in the IEEE 802.11-based network.

**Figure 5:**
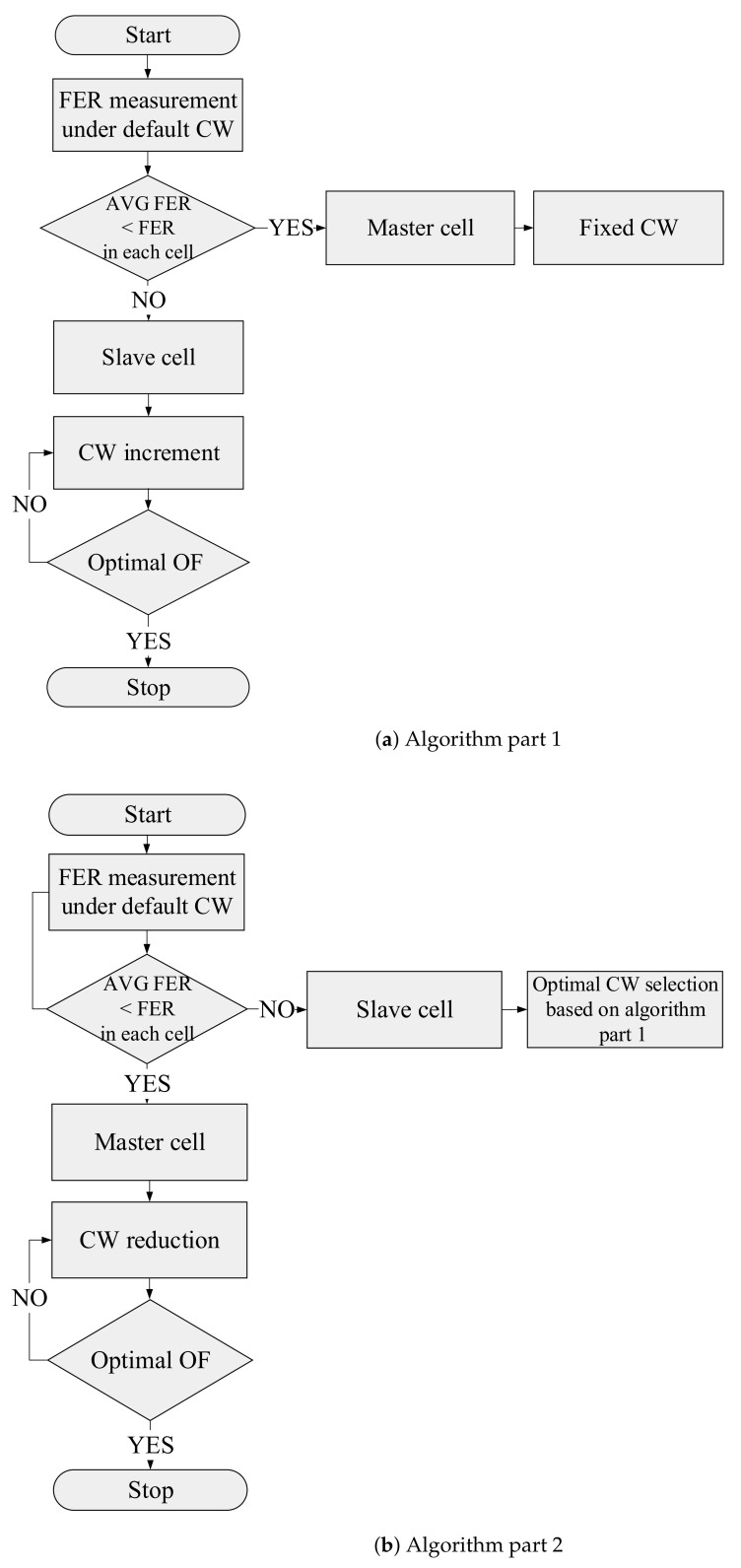
Flow graph of the AP coordination based optimization algorithm.

As the preliminary step to the algorithm, the CW is changed in all the cells to find the optimal CW combination for the network. The CW optimal value for each desired medical application is selected based on the level of QoS parameters and energy consumption per cell. Then the procedure of the algorithm starts by dividing the APs into two groups based on the Frame Error Rate (FER) per cell obtained values when the CW values are set to the standard values. The cells with a greater FER value than the average FER per cell are labeled as master cells; otherwise, they are labeled as slave cells. In the next phase, the CW values of master cells are assigned to the optimal CW values, which are obtained in the preliminary step, and the algorithm increases the CW values for slave cells according to Equation (1). For each set of CW combinations, the QoS parameters such as delay, PLR, and FER are analyzed, and if they meet the medical-grade QoS requirements, the algorithm will stop. Otherwise, the phase where the CW values are increased will repeat until these metrics meet the requirements. The flow graph in Figure 5a illustrates each stage of the procedure of the algorithm.

The second process of the algorithm, which is demonstrated in Figure 5b, starts by dividing the cells into two groups of master and slave cells, as explained in algorithm part 1. However, at this point, CW values are kept constant at the optimal CW values, which are obtained based on part 1, and the CW values on master cells shrink by a non-standard value gradually. In the end, for each set of CW combinations, the desired QoS parameters are analyzed. If they only meet the medical-grade QoS requirements, the algorithm will stop; otherwise, the phase where the CW values are decreased will repeat until these metrics meet the requirements.

### 4.2. Sleep/Wake-Up Mode

**Figure 6:**
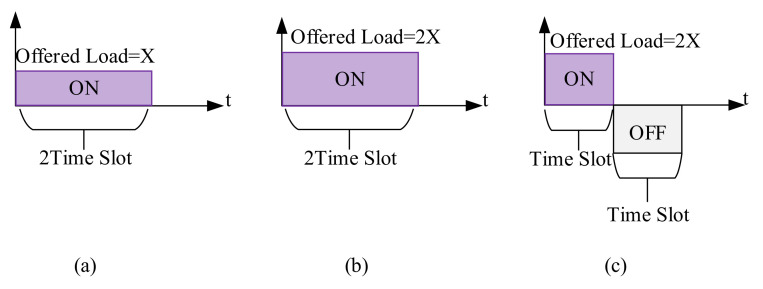
Sleep/Wake-up mode schematic. (**a**) Transmission before applying the sleep/wake-up technique, (**b**) offered load multiplication by factor X, (**c**) wake-up and sleep duration division by same factor X.

Additionally, we introduce a sleep/wake-up technique in the network. When the remaining energy of the network drops below a certain level, this method is triggered. It would considerably reduce the energy consumption of the non-AP stations associated with both slave and master APs while having a negligible impact on medical-grade QoS requirements. However, the greatest benefits are for the master cells, that otherwise had increased FER, which resulted in more energy being consumed in collisions. The schematic of this technique is illustrated in Figure 6.

We multiply the offered load by a factor of X (let us say 2, cf. Figure 6b). To transmit the same data along with the addition of the systematic sleep procedure, the wake-up duration and sleep duration for master cells are divided by the same factor X in a manner that the master can only transmit from A seconds to 1/X seconds (e.g., 0 to 0.5 s) and sleep from 1/X to 1/X + 1/X seconds (i.g., 0.5 to 1 s, cf. Figure 6c). Consequently, the slave is only allowed to transmit when the master is in sleep mode (i.g., 0.5 to 1 s). Since each second of transmission is specifically divided base on master and slave cells, A would be continuous with time. Furthermore, the designed algorithm is generic enough to have more than one set of master and slave cells (i.e., if X is 4, then two sets of Masters and Slaves could be used).

The proposed sleep/wake-up mode concept is adapted from the assertion that unnecessary wake-up duration is reduced by forcing a non-AP station to sleep according to its periodicity. According to this technique, the non-AP stations are set to partitions based on the BSS. Then, within each group, the AP has the permission to define a sleep/wake-up duration for each non-AP station to control their access to the channel and reduce the contention on the medium [59].

**Figure 7:**
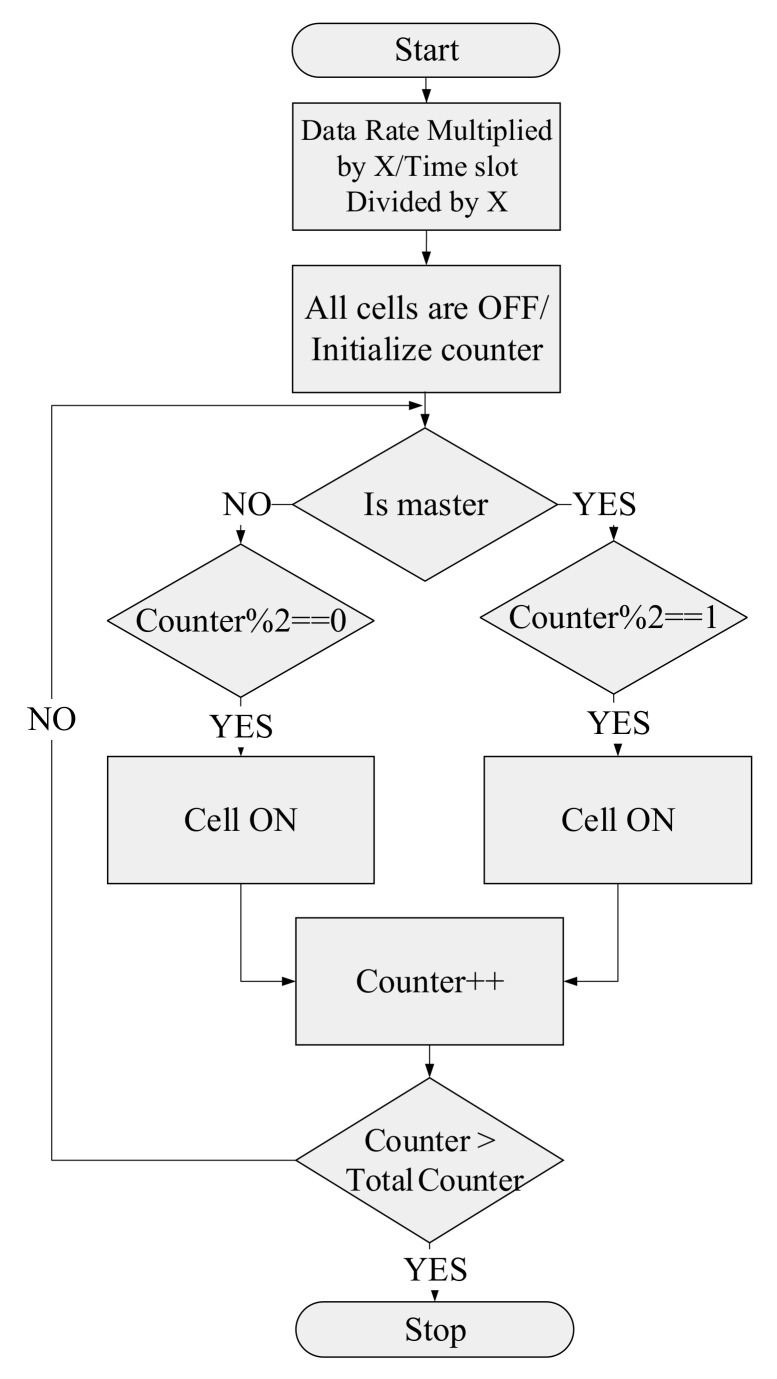
Flow graph of the Sleep/Wake-up mode algorithm.

In accordance with the proposed sleep/wake-up algorithm, in the first stage, when all the cells are in sleep mode, the data rate needs to multiply by the factor of X and time slot is divided by the factor of X, and the counter is initialized. In the next step, if the cell is selected as a master cell, it operates from A second to 1/X seconds (when the counter is an odd value) and is then sent to the sleep mode from 1/X seconds to 1/X + 1/X seconds (when the counter is an even value). In the case of the slave cell, the sleep duration corresponds to the master cell’s wake-up duration. This procedure continues until the algorithm’s counter reaches the total simulation time, and it will stop. The flow graph of the proposed sleep/wake-up method is illustrated in Figure 7.

Despite the fact that the defined sleep/wake-up method aims to reduce the network’s energy consumption, it mimics and addresses the intermittent communications challenge in the Wi-Fi environment. Intermittent communication becomes challenging in the bursty channel with a high level of interference or when there is not enough energy to keep the system powered up, where interruptions in communication are possible.

### 4.3. System Model

**Figure 8:**
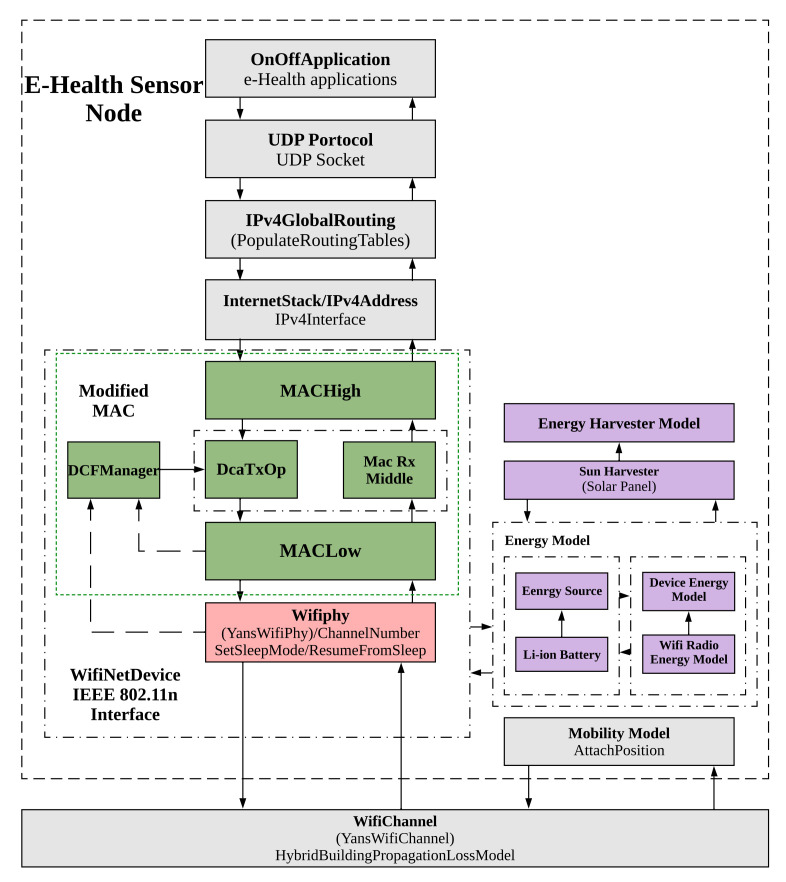
The layered structure of a sensor node (The modules in green, pink and purple represent the modified MAC layer, PHY layer, energy model, and energy harvester, respectively).

The structure of the ns-3 sensor node (non-AP station) is illustrated in Figure 8, in which different layers of IoT protocol stack along with energy-related modules are presented. As shown in this figure, our studies focus on the PHY layer, MAC layer, and energy-related modules illustrated in color. In contrast, the other layers of the IoT protocol stack, such as the network, transportation, and application layers, including the channel and mobility models, are in grayscale, meaning no changes are applied in this work.

The PHY layer, which is shown in pink, sets different transmission states of communication and the sleep/wake-up state for each station. The MAC layer presented in green is responsible for adjusting the EDCA values for each category, where our AP coordination technique is introduced. The energy-related modules illustrated in purple consist of three main parts: device energy model, Wi-Fi radio energy model, and energy source. The energy source considers different batteries, such as an RV battery, Li-ion battery, or even a capacitor. In addition, this module is responsible for setting the specific parameters for each type of energy source. The Wi-Fi radio energy module defines the consumed energy in each transmission state. Furthermore, it is responsible for calculating the network’s total energy consumption. In addition, the Wi-Fi radio energy module is installed on each station through the device energy model [60]. Apart from these modules, there is a solar energy harvester, which is designed for ns-3 [58]. However, this module does not exist in the official ns-3 versions. The ns-3 solar harvesting system is an accurate model which considers different aspects of the harvesting process. This system realistically designs a solar panel and mathematically models various characteristics of the sun, which have an impact on harvesting the energy. The actual amount of power that a solar panel harvests from sun radiation at a given time is obtained through Equation (2).

(2) $$P_{\mathrm{Sun}\;\mathrm{Harvester}}=\eta_{\mathrm{sc}}\;\times \;\eta_{\mathrm{DC}-\mathrm{DC}}\;\times \;D_{\mathrm{Panel}}\;\times \;I_{M}\left(t\right)$$

where $P_{\mathrm{Sun}\;\mathrm{Harvester}}$ is the total harvested power, $\eta_{\mathrm{sc}}$ and $\eta_{\mathrm{DC}-\mathrm{DC}}$ represent the solar cell efficiency and DC to DC converter efficiency, respectively. $I_{M}\left(t\right)$ is the insolation parameter, which is perceived by the surface of solar panel and is obtained through Equation (3).

(3) $$I_{M}\left(t\right)=I_{\mathrm{direct}}\left(t\right)\;+\;I_{\mathrm{diffused}}\left(t\right)$$

According to Equation (3), $I_{\mathrm{direct}}\left(t\right)$ and $I_{\mathrm{diffused}}\left(t\right)$ represent the direct radiation of the sun and diffused radiation of the sun, respectively. These parameters may vary during the year and depending on the position of the sun and the day time.

The general expression to obtain the energy consumption of wireless communication in IEEE 802.11 is defined through Equation (4) [61]:

(4) $$E_{\mathrm{Total}}=T_{\mathrm{Rx}}\;\times \;P_{\mathrm{Rx}}+T_{\mathrm{Tx}}\;\times \;P_{\mathrm{Tx}}+T_{\mathrm{Sl}}\;\times \;P_{\mathrm{Sl}}+T_{\mathrm{Id}}\;\times \;P_{\mathrm{Id}}$$

The power consumption of each state (reception, transmission, sleep, and idle) is the multiplication of the power consumption of that state to its corresponding duration.

### 4.4. Evaluation Metrics

Our system model is evaluated in terms of the following metrics.

#### 4.4.1. End-to-End Delay

This metric represents the average of the mean delay parameter for all the network stations. The mean delay parameter is considered when the source generates the frame until it reaches its destination. Thus, it includes delays due to transmission, queuing, and contention [62].

#### 4.4.2. Throughput

This metric refers to all the data frames that have been received successfully at the destination of the communication. This metric is obtained through Equation (5) [62].

(5) $$S=\frac{{\mathrm{Rx}}_{\mathrm{Bytes}}\;\times \;8}{\mathrm{Tx}{}_{\mathrm{Time}}}$$

where the ${\mathrm{Rx}}_{\mathrm{Bytes}}$ is the number of the received frames in bytes, and ${\mathrm{Tx}}_{\mathrm{Time}}$ is the duration between the last received frame and the first transmitted frame.

#### 4.4.3. FER

This metric is calculated through Equation (6). Since in IEEE 802.11, all the successfully received frames by the destination are acknowledged, to obtain the Frame Success Rate (FSR), we divided the number of acknowledged frames by the total transmitted frames [63].

(6) FER=1−FSR

#### 4.4.4. Collision Rate

The collision occurs when two or more stations try to send data frames over the shared channel simultaneously. This metric is calculated through Equation (7).

(7) $$Collision\;rate=\frac{{\mathrm{Rx}}_{\mathrm{error}}}{{\mathrm{Rx}}_{\mathrm{error}}+\frac{\mathrm{RxOk}}{2}}$$

where ${\mathrm{Rx}}_{\mathrm{error}}$ and ${\mathrm{Rx}}_{\mathrm{Ok}}$ represent the total number of the frames that have been received unsuccessfully and successfully, respectively. Since in a successful transmission ${\mathrm{Rx}}_{\mathrm{Ok}}$ is taken into account twice (one for the data frame and one for the ACK frame), to calculate the collision rate value, we need to divide ${\mathrm{Rx}}_{\mathrm{Ok}}$ by 2.

#### 4.4.5. PLR

This metric is calculated by Equation (8).

(8) PLR=1−PDR

where the Packet Delivery Ratio (PRD) is the number of delivered packets divided by the total number of sent packets [62].

#### 4.4.6. Fairness

This metric is defined through Jain’s fairness index (see Equation (9)), which determines the share of each station in the network resources [64]. This value is bounded between 0 and 1 (all stations have the same share of resources).

(9) $$Fairness=\frac{{\left(\sum_{i=1}^{n}S_{i}\right)}^{2}}{n\;\times \sum_{i=1}^{n}{S_{i}}^{2}}$$

where $S_{i}$ is the throughput of the ith station, and n represents the number of the stations in the network.

#### 4.4.7. Objective Function


(10) $$OF=\frac{\mathrm{Remaining}\;\mathrm{energy}}{\mathrm{Delay}\;\times \;PLR}$$

Since, in this paper, our target is to reduce energy consumption while maintaining the QoS requirement for medical applications, there is a need to define the level of QoS parameters for each medical application. For this reason, Table 4 is proposed based on the existing literature on real-time, emergency, and medical applications. Medical monitoring applications, along with video and telemetry alarm, are time-sensitive applications, while the Electronic Medical Record (EMR) is not a time-sensitive application. The ECG and Electroencephalogram (EEG) applications are considered applications with moderate latency, which means the end-to-end delay needs to be lower than 250 ms. In contrast, since a telemetry alarm is considered an emergency application, it requires a lower end-to-end delay (<100 ms). In EMR and video, this value can be higher(<400 ms). The required bandwidth for all medical applications remains the same (1 Mbps); however, in the case of video streaming, due to its high data rate, larger bandwidth is required. The PLR metric has to stay under 10% for all the medical applications and emergency services. However, in the case of video streaming, this value has to reduce to 5%. In Section 6, we will validate our analysis and obtain results based on Table 4.

## 5. Simulation Setup

To evaluate the performance of the proposed system model, we implement a dense solar-based Wi-Fi network in a field hospital in the ns-3 simulation environment. In this section, we explain the simulation setup environment under designated conditions.

### 5.1. Network Scenario Definition and Assumptions

**Figure 9:**
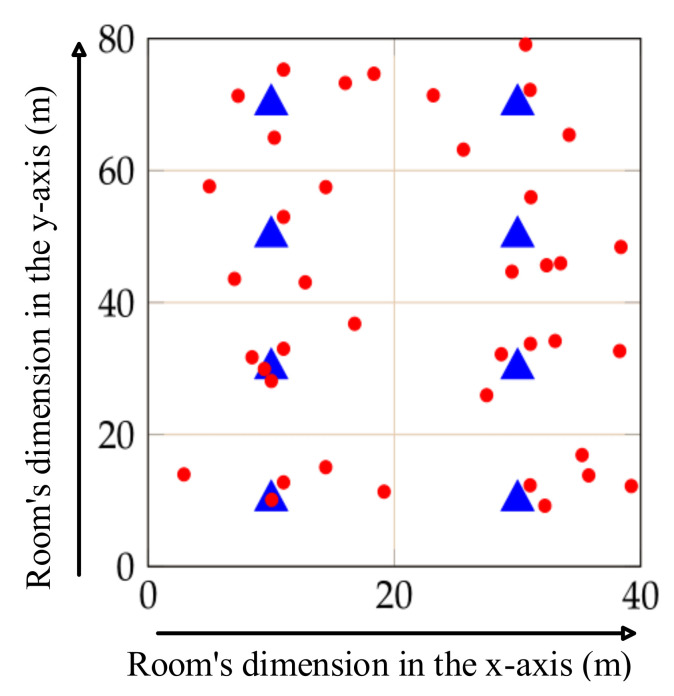
Layout of the Wi-Fi deployment in field hospital.

We consider a field hospital to be the simulation environment, where the relevant propagation loss model is the hybrid buildings propagation loss. The field hospital has one floor of 3 m height off the floor. The type of the hospital is considered an office-type building. The area of the field hospital is 40 m × 80 m, and the size of each room inside the field hospital is defined as 20 m × 20 m. The rooms are separated via wooden walls, and the external walls are considered concrete with windows. We locate one AP in the center of each room and associate the *n* number of non-AP stations to each AP. The stations are arranged in a circular pattern around the AP in each room, ranging in the distance from 1 to 10 m, and connect with the AP in the uplink direction. In this paper, the transmission performance is based on the IEEE 802.11n amendment. This represents a worst-case scenario since IEEE 802.11n uses the 2.4 GHz frequency band, which suffers from interference that impacts its performance, particularly in dense environments. In each set of simulations, to evaluate the performance of the network model, we consider the network size of five non-AP stations associated with each AP. The layout of the deployment when *n* = 5 is illustrated in Figure 9, where the brown lines represent the internal walls, and the black lines demonstrate the external walls. Moreover, the blue triangles and red circles represent APs and non-AP stations, respectively. In addition, the depicted numbers on the X and Y dimensions, represent the length of each room corresponding to these dimensions.

Each station is equipped with four different e-Health applications (ECG, EEG, EMR, and Telemetry alarm) in the simulations. The demonstration of the physical and default EDCA MAC layers parameters and medical traffic characteristics for the simulation are detailed in Table 5, Table 1 and Table 6, respectively.

According to the priority of each e-Health application, specific access categories are defined for them. Telemetry alarm has the highest priority (AC${}_{\mathrm{VO}}$) among the selected applications in these network evaluations, and ECG, EEG, and EMR have the lowest priority (AC${}_{\mathrm{BE}}$). To calculate the ON-OFF period in the case of the telemetry alarm, since the traffic pattern shows 3.6 events per hour and each event duration is 1 s, we divide the number of events per hour to find the probability of the ON period (0.001) and the probability of the OFF period is 0.999. In the case of EEG [70] the probability of the OFF period is defined as 0.71; consequently, the ON probability is 0.29. In the case of EMR, since it represents the medical file transferring, it has a probability of 0.05 for the ON period and 0.95 OFF period. This means that file transmissions are not frequent. In the end, in the case of ECG, the probability of the ON period is defined as 0.65, and the probability of OFF duration is 0.35 [71]. Since ECG and EEG are both telemonitoring applications, the traffic type for ON and OFF is defined as the Constant Bit Rate (CBR). However, telemetry alarms and EMR have exponential traffic types (cf. Table 1).

**Table 5:** Physical layer parameters for simulation.

Parameter	Value
Wireless Standard	IEEE 802.11n
Frequency band	2.4 GHz
Physical transmission rate	MCS 5 for data frames
Propagation loss model	Hybrid building propagation loss
External Wall penetration loss	7 dB
Internal Wall penetration loss	4 dB
Transmission power	16 dBm
Energy detection threshold	−62 dBm
CCA mode1 threshold	−82 dBm
Guard interval	Short
Channel bandwidth	20 MHz
Channel Number	1
Aggregation	Disable
Stations per AP	5

**Table 6:** Traffic characteristics in the simulation study.

Traffic Type	ECG	EEG	EMR	Telemetry Alarm
**Access Category**	**BE**	**BE**	**BE**	**VO**
Traffic model	ON-OFF	ON-OFF	ON-OFF	ON-OFF
	(0.650–0.350)	(0.29–0.71)	(0.05–0.95)	(0.001–0.999)
	CBR [72]	CBR [40]	Exponential [40]	Exponential [40]
Data rate	12 kbps [71]	32 kbps [73]	4.1 Mbps [40]	5 kbps [40]
Packet size (Bytes)	147 [40]	155 [73]	1528 [40]	668 [74]

Moreover, each non-AP station is equipped with a solar panel with the dimension of 17 ${\mathrm{cm}}^{2}$ whose size is matched with a remote blood oxygen monitoring [75]. Furthermore, the geographic coordination of the panel is set to the Barcelona city with a latitude of 41.3851${}^{\circ }$, longitude of 2.1734${}^{\circ }$, and altitude of 12 m above the sea. To obtain the energy consumption of each state of transmission, the current consumption of these states is defined according to to [76]. Additionally to the solar panel, each non-AP station is equipped with a Li-ion battery as a source of energy, whose characteristics are listed in Table 7.

Moreover, each non-AP station is equipped with a solar panel with a dimension of 17 ${\mathrm{cm}}^{2}$ whose size is matched with a remote blood oxygen monitoring. Furthermore, the geographic coordination of the panel is set to the Barcelona city with a latitude of 41.3851${}^{\circ }$, longitude of 2.1734${}^{\circ }$, and altitude of 12 m above the sea. In addition to the solar panel, each non-AP station is equipped with a Li-ion battery as a source of energy, whose characteristics are listed in Table 7. To obtain the energy consumption of each state of transmission, the current consumption of these states is defined according to [76].

This setup permits us to examine the performance of our proposed algorithm by varying the CW value as a MAC parameter together with the offered sleep/wake-up method.

Next, the energy consumption based on the different communication states is analyzed.

### 5.2. Energy Consumption of Each Transmission State

To completely understand the total energy consumption in each transmission state of the communication, we analyze the network performance under saturated traffic (there is always a frame to transmit) and non-saturated traffic with and without applying the sleep/wake-up method.

**Figure 10:**
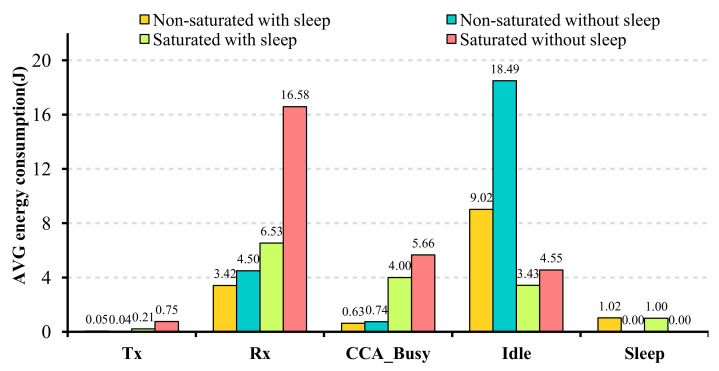
The idle and Rx states are responsible for most of a node energy consumption.

According to Figure 10, which demonstrates the energy consumption of each state of transmission for the selected scenarios, in the case of saturated traffic (with/without sleep), the most consuming energy is the reception state. The reason behind it is that in Wi-Fi standard communications, the stations always sense the shared medium and receive the preamble frame of the communications of all the contenders. Then they decode the preamble frames only if they are meant for those stations. This procedure has a considerable impact on energy consumption under saturated conditions. In contrast to the saturated condition, in the case of the non-saturated network condition, since most of the time stations are in the idle state, almost 80% of the consumed energy belongs to this state (cf. Figure 10). In addition, Figure 10 coveys that applying sleep/wake-up mode reduces the energy consumption from 23.77 J to 14.14 J and from 27.55 J to 15.17 J in non-saturated and saturated scenarios, respectively. In the case of the saturated scenario, although the energy consumption of all the transmission states reduces, this parameter is reduced to more than half for the reception state. In the case of the non-saturated scenario, the most consuming energy state is the idle state, whose energy consumption reduce to half by applying the sleep/wake-up method.

## 6. Performance Evaluation and Discussion

In this section, through extensive simulations, we assess the performance of our AP coordination-based optimization algorithm in a solar-based dense Wi-Fi network implemented in a field hospital. As described in Section 4, to find the most proper CW combination for each medical application, the proposed simulations run in the selected environment for each application individually based on three steps (i) CW changes on all cells of the network, (ii) CW changes only on slave cells, (iii) CW changes only on master cells. Then, we apply the sleep/wake-up mode to improve the network performance regarding energy consumption. The analysis is based on the PLR and end-to-end delay as the medical-grade QoS feature, FER, and energy consumption. Finally, we demonstrate the importance of the energy harvester implementation in the network.

### 6.1. Adaptation to the CW Changes on All the Cells

Although IEEE 802.11 standard defines specific CW values for each AC, these values may need to vary based on the network conditions for each type of traffic. The selection of proper CW values has an essential impact on the network’s performance. For instance, the network will suffer from a long delay if the CW values are selected as very large values. In contrast, in the case of selecting a very small value for CW, the collision rate will increase. Since we consider three different medical applications with distinct traffic models, we must first find the most proper CW combination for each application in these simulations.

For this reason, we first increase the CW combinations from default values (the standard combination for AC${}_{\mathrm{BE}}$) to cases 1, 2, 3, and 4 in all the cells. Although different possible combinations can be defined, to summarize the results, we consider four cases in our simulations that are listed in Table 8. In these simulations, five non-AP stations are associated with each AP during 30 s of the simulation run. We repeat the exact simulation 10 runs to obtain more accurate results.

#### 6.1.1. CW Changes under ECG Application

**Figure 11:**
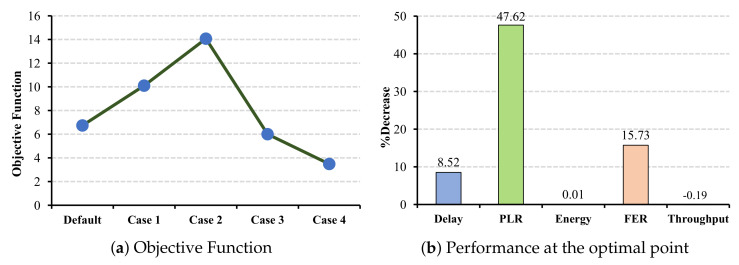
ECG QoS metrics and energy consumption under CW changes for all cells.

Figure 11a shows the objective function in the case of ECG application when CW changes in all the cells. According to the objective function, the most improvements in the network belong to case 2. We can see that the objective function decrease in cases 3 and 4 due to the large CW combinations, which delay the transmission. The PLR parameter can benefit from increasing the CW combination. However, selecting a large CW combination, such as the ones in cases 3 and 4, increases the packet loss, which causes a reduction in the objective function. The reason for this is that all the non-AP stations are delayed for a longer duration.

In contrast to the delay and PLR parameters, energy consumption and FER can benefit from increasing the CW combination, where FER has a significant reduction (47.62%) among all other metrics (cf. Figure 11b). Although the energy consumption is decreased, this reduction is almost 0.01% and is negligible. The reason for this is that selecting a larger CW value reduces the collision ratio and FER, which both impact energy consumption. Nevertheless, to choose the most proper combination and consider all four metrics together, and based on the objective function, case 4 has the worst impact on the network performance, and case 2 has the most suitable case for ECG application. In addition to the QoS metrics, the results indicate a possible but slight improvement in throughput parameters.

#### 6.1.2. CW Changes under EEG and EMR Applications

**Figure 12:**
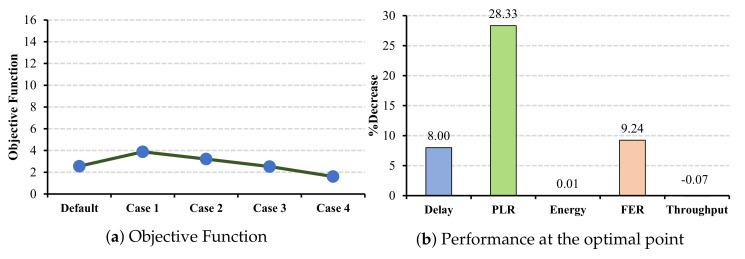
EEG QoS metrics and energy consumption under CW changes for all cells.

**Figure 13:**
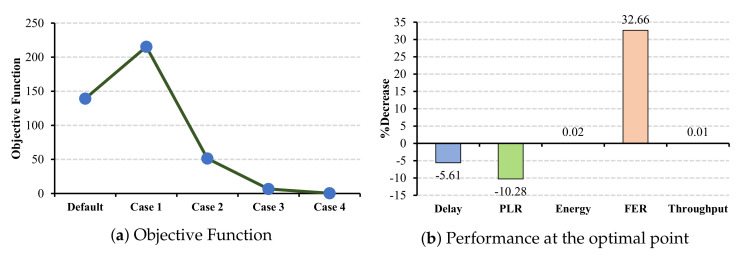
EMR QoS metrics and energy consumption under CW changes for all cells.

According to the objective functions for EEG and EMR applications, which are illustrated in Figure 12a and Figure 13a, the optimal case in both applications is case 1. Moreover, the objective functions indicate that case 5 has the worst impact on the network performance. The reason for this is that in case 5, the network suffers from large delay and PLR.

As shown in Figure 12b, all the metrics in the optimal case are improved even though there is a slight increase in the throughput parameter. However, in the case of EMR application (cf. Figure 13b), although FER is decreased by 32.66%, which causes a slight reduction in the energy consumption, PLR and delay are increased by 10.28% and 5.61%. Nevertheless, since the increasing percentage in PLR and delay are drastic, these two metrics stay below the QoS restrictions for EMR application. By comparing the FER in Figure 12b and Figure 13b, we convey that a longer delay causes more reduction in FER and energy consumption. Therefore, in case 4, with a larger CW combination, there is a huge improvement in network performance in terms of FER and energy consumption. However, we cannot consider this case to be the optimal point due to its large PLR and delay that cause a drastic reduction in the objective function. Thus, we need to take into consideration the best value of all the metrics simultaneously. For this reason, case 2, with the least PLR and delay for both applications, has the best network performance under this CW combination. As objective functions demonstrate, this case is considered to be the optimal CW combination.

We observe that the proper CW combination for ECG application is larger than the CW combination for EEG and EMR applications. The reason for that is the traffic type of this application with a larger probability for the ON period compared to EEG and EMR applications.

### 6.2. Adaptation to CW Changes on Slave Cells

In the next step, we evaluate the network’s performance when the master cells keep their CW values constant based on the previous step (optimal combination) and request the slave cells to increase their CW values.

#### 6.2.1. CW Changes under ECG Application

**Figure 14:**
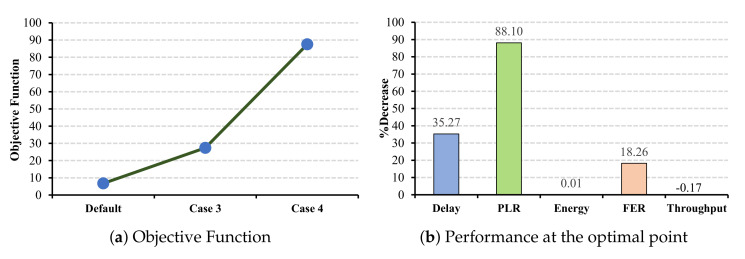
ECG QoS metrics and energy consumption under CW changes for slave cells.

Increasing the CW values on the slave cells allows the master cells to improve their medical-grade QoS metrics and energy consumption performance by starting the transmission faster than the slave cells. The larger the CW combination selected, the more the network benefits. As shown in Figure 14a, by comparing the obtained results to the default case, the maximum objective function belongs to case 4. However, similar to the previous results, the percentage reduction in energy consumption is negligible.

#### 6.2.2. CW Changes under EEG and EMR Applications

**Figure 15:**
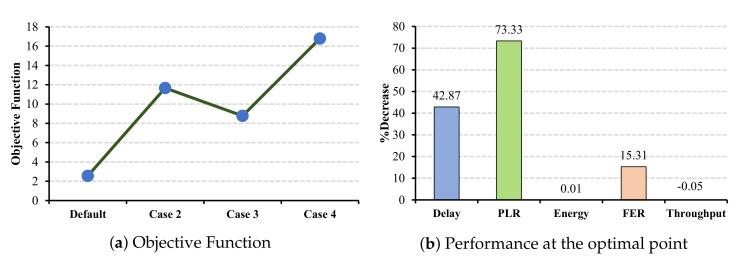
EEG QoS metrics and energy consumption under CW changes for slave cells.

**Figure 16:**
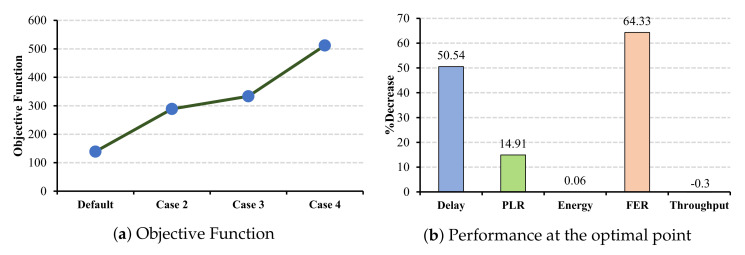
EMR QoS metrics and energy consumption under CW changes for slave cells.

Similar to the ECG application, in the case of EEG and EMR applications, all the QoS metrics benefit from a larger CW selection. Increasing the CW values for EEG application has the most impact on the PLR parameter by decreasing 73.33% from the standard value (cf. Figure 15b), and the EMR application has the most impact on the FER by a 64.32% decrease (cf. Figure 16b).

We observe that to increase the performance of the master cells and give more opportunity for them to transmit faster with less collision, the CW values in the slave cells need to be larger than the CW values in the master cells. However, selecting a minimal CW value for the master cell increases the collision among them and degrades their performance. For this reason, we need to find the most proper CW combination for master cells as well.

### 6.3. Adaptation to CW Changes on Master Cells

As mentioned previously, by increasing the CW value on slave cells and delaying their transmissions, the master cells will have more opportunities to start transmission. Additionally, shrinking the CW values on master cells will offer them even more opportunities to start the transmissions faster. However, this can increase the collision rate among master cells with smaller CW values. Therefore, there is an optimal CW value for master cells to increase their transmission opportunity. In this step, the slave cells keep the CW values constant based on the previous step to case 4 for all three applications and gradually shrink the CW values on the master cells according to Table 9.

#### 6.3.1. CW Changes under ECG and EEG Applications

**Figure 17:**
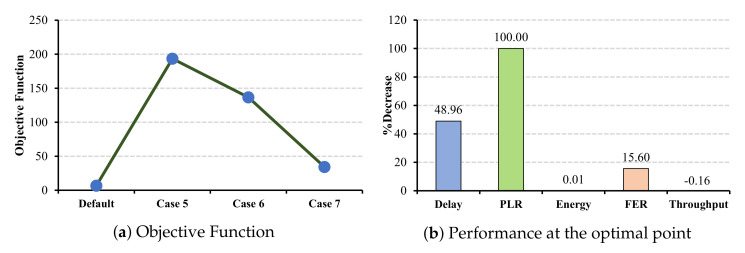
ECG QoS metrics and energy consumption under CW changes for master cells.

**Figure 18:**
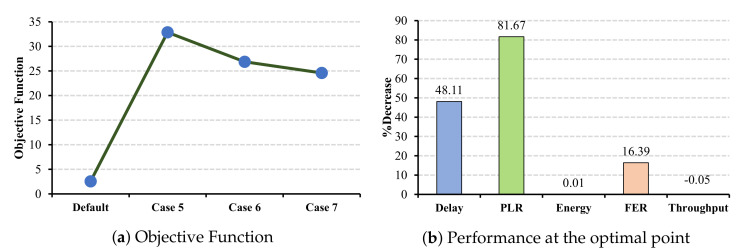
EEG QoS metrics and energy consumption under CW changes for master cells.

In this set of simulations, the CW values in master cells shrink from case 5 to case 7. In the ECG application, as shown in Figure 17a, case 5 is the optimal selection, where the PLR benefits more than other metrics and decreases by 100% (cf. Figure 17b). However, this reduction cannot be observed in energy consumption, where the decrease percentage is only 0.01%. Similarly, in the EEG application, as the objective function demonstrates, case 5 is the optimal CW combination, where the delay and FER benefit more than other metrics (cf. Figure 18b).

In this combination, CW values in slave cells stay large enough to allow master cells to start communication before them. The CW values in master cells are small enough to make their transmission faster while maintaining the level of medical-grade QoS requirements below the restrictions.

#### 6.3.2. CW Changes under EMR Application

**Figure 19:**
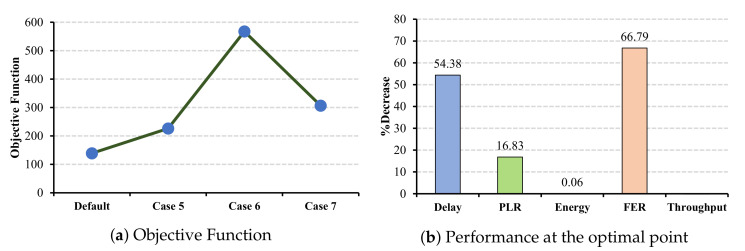
EMR QoS metrics and energy consumption under CW changes for master cells.

In contrast to the ECG and EEG applications, in EMR application case 6 demonstrates the maximum decrease in delay and PLR (cf. Figure 19b and while having an acceptable level of decrements in FER 66.79%.

The analysis based on the obtained results indicates that, although the medical-grade QoS requirement is met through our AP coordination-based algorithm, the energy consumption reduction compared to the standard CW combination is not as much as QoS metrics. For this reason, in the following subsection, we will introduce the sleep/wake-up mode to reduce the network’s energy consumption with a focus on master cells.

### 6.4. Sleep/Wake-Up Mode with CW Changes

As we explained in Section 4, to reduce the network’s total energy consumption, we introduce a sleep/wake-up mode to the network with the optimal CW combinations in both master and slave cells. According to this method, for the case of simplicity, the wake-up duration and sleep duration for the master cell are defined as 0 s–0.5 s and 0.5 s–1.0 s, respectively. In this method, while the master cell is in sleep mode, the slave cell has permission to transmit, and during the wake-up duration of the master cell, it is sent to sleep mode. To avoid losing frames and degrading the network’s performance in terms of throughput for each medical application, we double the data rate. In this case, in the ON period (0.5 s), each application transmits the same amount of data sent before.

**Figure 20:**
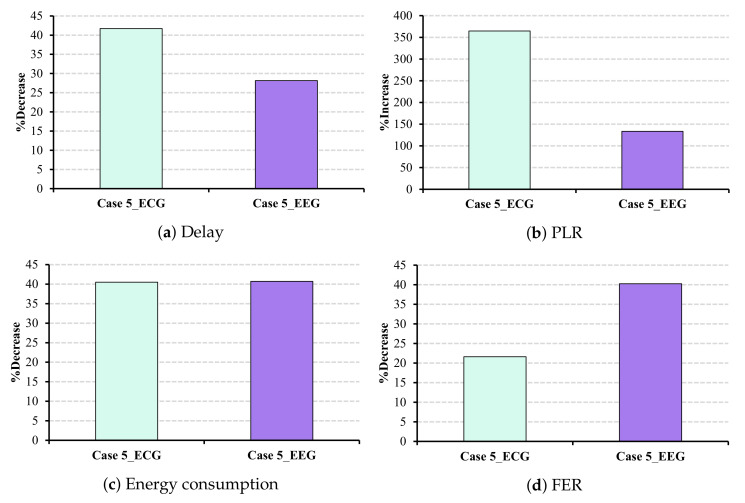
Desired QoS metrics and energy consumption under CW changes with sleep/wake-up mode.

Compared to the obtained results of the standard without sleep/wake-up mode, although PLR in both ECG and EEG applications increases, it still stays below the QoS restrictions. Nevertheless, other QoS parameters such as delay and FER improve, and energy consumption experiences a considerable reduction of almost 40% in both applications (cf. Figure 20c).

### 6.5. Impact of Energy Harvester

**Figure 21:**
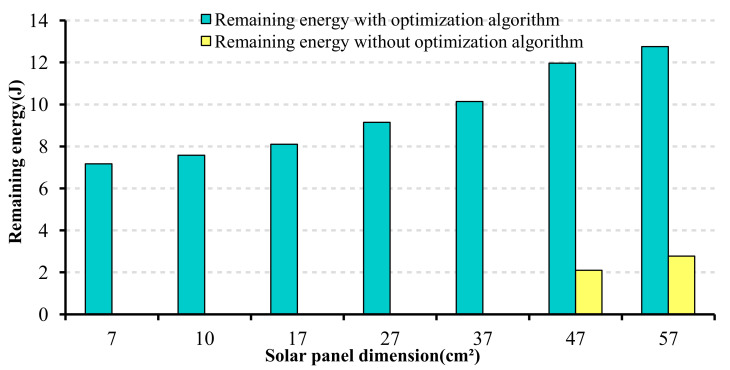
Impact of the proposed algorithm on the selection of the solar panel.

As we explained throughout the paper, IoT systems benefit from deploying energy harvesting technologies, specifically when providing a reliable energy source for devices. Thus, to reveal the critical role of the energy harvester in our simulations, we compared the network’s remaining energy in two scenarios while changing the size of the solar panel. On the one hand, with the implementation of the proposed algorithm, and on the other hand, without the proposed algorithm. The results indicate that in the network without the algorithm’s implementation, larger panel size is required to provide enough energy to the network system (the smallest possible dimension, in this case, is 47 ${\mathrm{cm}}^{2}$). However, when applying the algorithm, it is possible to reduce the size of the panel from 47 ${\mathrm{cm}}^{2}$ to 7 ${\mathrm{cm}}^{2}$, which means the harvested energy from a panel size of 7 ${\mathrm{cm}}^{2}$ will be sufficient to keep the system to powered up. This comparison, which is illustrated in Figure 21, conveys the effectiveness of the proposed algorithm on the solar panel size reduction, and consequently, the feasibility of the cooperation of the energy harvesting technologies and Wi-Fi-based IoT systems.

### 6.6. Discussion

Results show that the EDCA CW selection should be adapted for different medical applications. For instance, in the presented work, since the traffic characteristics of the considered medical applications (ECG, EEG, and EMR) are varying, there is not a unique valid CW combination. Thus, there is an optimal CW combination within the same AC for each application. Furthermore, according to the results, the network performance in terms of medical-grade QoS, in this case, delay and PLR, can be improved by introducing AP coordination within IEEE 802.11 standard. In this context, first, the transmission in slave cells is delayed by increasing their CW values, then the CW values shrink in master cells to give them more opportunity to start the transmission. However, there is an optimal value for CW values in both master and slave cells to not increase the collision rate probability by decreasing the CW and delay the slave a lot by increasing this value. Additionally, the proposed sleep/wake-up mode considerably reduces energy consumption in medical applications without violating the QoS restrictions. Finally, we indicate the importance of the energy harvester in the system and the effectiveness of our proposed algorithm, which can reduce the dimension of the required solar panel.

## 7. Conclusions and Future Works

This paper presents a novel e-Health-oriented communications system, simulated in ns-3, where the cooperation of energy harvesting technologies with Wi-Fi-based IoT systems is possible. We introduced an AP coordination-based optimization algorithm in the MAC layer that encounters the optimal CW combination in both master and slave cells separately to improve the non-AP station’s performance associated with the master APs in terms of medical-grade QoS. The results indicate that the proposed algorithm can improve the level of the QoS metrics for considered applications at most 80% (different for various applications and metrics). In addition, the proposed algorithm aligned with the sleep/wake-up method introduces a reduction of more than 40% in the network’s energy consumption while maintaining the QoS metrics below the restriction level. We consider that this paper could shed light on enabling the integration of energy harvesting in IoT systems. In future work, this study can be expanded in terms of adapting the proposed algorithm to other MAC layer parameters rather than just CW. In addition, to covey a more profound analysis, the per non-AP station evaluation for a more dense network can be conducted.

## Figures and Tables

**Table 1:** Default EDCA ACs parameters.

**Access Category**	**CW${}_{\min}$**	**CW${}_{\max}$**	**AIFSN**	**TXOP**
VO	7	15	2	1.5 ms
VI	15	31	2	3.0 ms
BE	31	1023	3	0.0 ms
BK	31	1023	7	0.0 ms

**Table 2:** Relevant energy harvesting for MIoT system [4].

Energy Source	Energy Harvester	Power Density
Sun radiation	Photo-voltaic cell	10 µW/${\mathrm{cm}}^{3}$
Artificial light		100 mW/${\mathrm{cm}}^{3}$
Radio Frequency	Wireless energy	0.1 µW/${\mathrm{cm}}^{2}$
	harvester	300 µW/${\mathrm{cm}}^{2}$
Heat	Thermocouple	40 µW/${\mathrm{cm}}^{2}$
		50 mW/${\mathrm{cm}}^{2}$
Human body motion	Piezoelectric	0.021 µW/${\mathrm{mm}}^{3}$
Vibration		2 W/${\mathrm{cm}}^{3}$

**Table 3:** Features comparison of related work and our proposal.

	Properties	Wireless Communication ^1^	MAC Modification	AP Coordination	Sleep/Wake-Up Deployment	Energy Harvester	QoS Support	Dense Deployment
Studies								
Son et al. [40]		Wi-Fi	✓	✗	✗	✗	✓	✗
Tian et al. [41]		Wi-Fi	✓	✗	✗	✗	✓	✗
Syed et al. [42]		Wi-Fi	✓	✗	✗	✗	✓	✓
Ahmed et al. [43]		Wi-Fi	✓	✗	✓	✗	✓	✓
Ali. et al. [44]		Wi-Fi	✓	✗	✓	✗	✓	✓
Ali. et al. [45]		Wi-Fi	✓	✗	✗	✗	✓	✓
Filoso et al. [46]		Wi-Fi	✓	✗	✗	✗	✓	✓
Malche et al. [47]		BLE	✗	✗	✓	✗	✗	✓
Sheela et al. [48]		Wi-Fi	✗	✗	✗	✗	✗	✓
Fafoutis et al. [49]		Wi-Fi	✓	✗	✓	✓	✗	✗
Lin et al. [50]		Wi-Fi	✓	✗	✓	✓	✗	✓
Shafique et al. [51]		Wi-Fi	✗	✗	✗	✓	✗	✓
Blobel et al. [52]		Wi-Fi	✓	✗	✓	✓	✗	✓
Kim et al. [53]		Multiple	✓	✗	✓	✓	✓	✗
Sarang et al. [54]		Multiple	✓	✗	✓	✓	✓	✗
Kim et al. [56]		Multiple	✓	✗	✗	✓	✗	✓
Naderi et al. [55]		Multiple	✓	✗	✗	✓	✗	✗
Guntupalli et al. [57]		Multiple	✓	✗	✓	✓	✗	✓
Our proposal		Wi-Fi	✓	✓	✓	✓	✓	✓

^1^ In multiple studies the focus of the authors is on the CSMA/CA as the channel access mechanism. Since this mechanism can be used in different wireless communication technologies such as Wi-Fi, Zigbee, LoRa (class C devices), and active RFID, we convey it as multiple wireless communication technologies.

**Table 4:** Quality of service requirements for e-Health applications.

Application Type	QoS Parameters				
	End-to-End Delay (ms)	Required Bandwidth (Mbps)	Packet Loss Ratio (%)	Jitter (ms)	Sensitivity to Context
ECG [40,65,66]	<250	1	<10	25	✓
EEG [40,65,66]	<250	1	<10	25	✓
EMR [40,67,68]	<300	1	<10	30	✗
Telemetry alarm [40,65]	<100	1	<10	25	✓
Video [69]	150–400	2	<5	30	✗

**Table 7:** Energy-related parameters.

Parameter	Value
Panel dimension [58]	17 ${\mathrm{cm}}^{2}$
Panel latitude [58]	41.3851${}^{\circ }$
Panel longitude [58]	2.1734${}^{\circ }$
Panel altitude [58]	12.000 m
Harvesting update interval [58]	0.100 s
Initial energy [77]	100.000 J
Initial voltage [77]	3.200 v
Nominal voltage [77]	4.000 v
Exponential voltage [77]	4.000 v
Rated capacity [77]	0.950 Ah
Nominal capacity [77]	1.600 Ah
Exponential capacity [77]	0.200 Ah
Internal resistance [77]	0.035 Ω
Minimum threshold voltage [77]	3.000 v
Idle current [76]	0.233 A
Transmission current [76]	0.466 A
Reception current [76]	0.300 A
Sleep current [76]	0.020 A
CCA_Busy [76]	0.273 A

**Table 8:** Label adaptation of CW combinations to find the optimal point.

Combination of CW${}_{\min}$	Adapted Label in the Case of
and CW${}_{\max}$	CW Changes in All the Cells
31–1023	Default
63–1055	case 1
127–1119	case 2
255–1247	case 3
511–1503	case 4

**Table 9:** Label adaptation of CW combinations in master cells.

Combination of CW${}_{\min}$ and CW${}_{\max}$	Adapted Label in the Case of CW Changes in Master Cells
31–1023	Default
123–1116	case 5
119–1112	case 6
115–1108	case 7

## Data Availability

Not applicable for this study.
